# Single-Dose Liposomal Amphotericin Plus Fluconazole and Flucytosine for Cryptococcal Meningitis at a US Public Hospital

**DOI:** 10.1001/jamanetworkopen.2025.53552

**Published:** 2026-01-21

**Authors:** Devin Clark, Javier Barranco-Trabi, Irene Goo, Maria Chyz, Grace Manchala, Kusha Davar, Sarah Freling, Noah Wald-Dickler, Rachel Baden, Brad Spellberg

**Affiliations:** 1 Division of Infectious Diseases, Los Angeles General Medical Center, Los Angeles, California; 2 Department of Medicine, Keck School of Medicine of the University of Southern California, Los Angeles; 3 Hospital Administration, Los Angeles General Medical Center, Los Angeles, California

## Abstract

**Importance:**

Cryptococcal meningitis (CM) is a significant cause of mortality and morbidity in people with HIV. Standard induction therapy for CM—amphotericin with flucytosine—is associated with significant toxic effects.

**Objective:**

To assess mortality and CM recurrence outcomes of the AMBITION protocol (single-dose liposomal amphotericin with 2 weeks of fluconazole and flucytosine) for treatment of CM in a high-resource setting.

**Design, Setting, and Participants:**

This pre-post cohort study conducted at Los Angeles General Medical Center, a large safety net hospital (80% Medicaid-covered, 5% uninsured), included people with HIV treated for CM with the AMBITION protocol from August 1, 2022, to July 31, 2024, and a control population receiving a guideline-compliant, daily amphotericin-based regimen from August 1, 2020, to July 31, 2022. Exclusion criteria included age less than 18 years, alternative diagnosis, transfer during induction, and death within 48 hours of admission.

**Exposures:**

AMBITION protocol with single infusion of liposomal amphotericin (10 mg/kg) and 14 days of high-dose fluconazole (1200 mg/d) and flucytosine (100 mg/kg/d). The control group was treated with lipid-based amphotericin (3-5 mg/kg/d) and flucytosine (100 mg/kg/d) for 14 days.

**Main Outcome and Measures:**

The primary end point was treatment success, defined as a composite of survival without recurrence or severe adverse events at 90 days.

**Results:**

A total of 60 eligible patient episodes were identified during the study period (54 [90.0%] among males; median patient age, 40 [IQR, 34-51] years). In all, 34 patients (56.7%) were treated with the AMBITION protocol, compared with 26 (43.3%) receiving the control regimen. Loss to follow-up by 90 days occurred in 6 patients in the control group (23.1%) and 9 intervention patients (26.5%) (*P* = .76). Of patients with available data, the composite outcome occurred in 19 of 25 patients receiving the AMBITION protocol (76.0%) compared with 7 of 20 patients in the control group (35.0%) (*P* = .006). The difference in composite outcome rates was driven by fewer severe adverse events in the AMBITION arm (7 [20.6%] vs 16 [61.5%]; *P* = .001). Additionally, patients receiving the AMBITION protocol had fewer patient-directed discharges (4 [11.8%] vs 9 [34.6%]; *P* = .03).

**Conclusions and Relevance:**

In this pre-post study of HIV-associated CM, fewer severe adverse effects were seen in patients receiving the AMBITION protocol compared with a daily amphotericin-based regimen, with no difference in mortality or recurrence. There were also fewer patient-directed discharges, suggesting more patient-centered care. These findings support broader adoption of the AMBITION protocol in high-resource settings.

## Key Points

**Question:**

What are the outcomes of the AMBITION protocol (single-dose liposomal amphotericin with 2 weeks of fluconazole and flucytosine) compared with guideline-compliant treatment for cryptococcal meningitis (CM) among patients with HIV in high-resource settings?

**Findings:**

In this cohort study of 60 patient episodes, the AMBITION protocol had similar mortality and CM recurrence rates compared with guideline-compliant treatment, with significantly fewer adverse events and less frequent patient-directed discharges.

**Meaning:**

The findings support broader adoption of the AMBITION protocol for CM in patients with HIV.

## Teaser

This cohort study explores survival and recurrence outcomes of the AMBITION protocol (single-dose liposomal amphotericin with 2 weeks of fluconazole and flucytosine) vs standard protocol for cryptococcal meningitis in patients with HIV.

## Introduction

Cryptococcal meningitis (CM) remains a significant cause of HIV-related morbidity and mortality, even in resource-rich settings. Although expanded HIV screening and antiretroviral therapy access have led to decreased rates of all opportunistic infections, CM continues to be a leading cause of death among people with HIV worldwide.^1^ For decades, the standard induction therapy for CM in resource-rich areas has been a prolonged inpatient course of daily amphotericin in combination with flucytosine for at least 14 days.^2,3^ This regimen is associated with significant toxic effects, including electrolyte abnormalities, nephrotoxicity, and infusion reactions. It requires close monitoring and extended hospitalization, which can be particularly burdensome on patients with employment constraints, caregiving responsibilities, or other competing social or economic needs. For some patients, the rigid recommendation for prolonged hospitalization may lead to discord with treating practitioners, further exacerbating health disparities.

The AMBITION randomized clinical trial (RCT) introduced a less toxic and primarily oral alternative protocol for the treatment of CM, using a single high-dose infusion of liposomal amphotericin B followed by 14 days of high-dose fluconazole and flucytosine.^4^ The trial showed that this regimen was noninferior to the protocol previously recommended by the World Health Organization (WHO) for resource-limited settings (7-day conventional amphotericin deoxycholate B and flucytosine followed by 7-day high-dose fluconazole monotherapy).^5^ In addition, there were significantly fewer grade 3 or 4 adverse events in the experimental vs the control arm (50% vs 62.3%; *P* < .001). These results prompted the WHO to update recommendations, identifying the AMBITION protocol (treatment with single-dose liposomal amphotericin and 2 weeks of fluconazole and flucytosine) as the preferred induction regimen,^6^ and the US National Institutes of Health has added it as an alternative option.^7^ However, the Infectious Diseases Society of America has not updated its guidelines since 2010, and concerns persist regarding the extrapolation of the results of a study conducted in low- and middle-income countries (LMICs) to higher-resource settings.^3^

Thus, despite its promise, there has been limited uptake of the AMBITION protocol in the US.^8^ Several concerns have been raised regarding its applicability. The mortality rate in the AMBITION trial far exceeded the rates seen in resource-rich settings, so it is possible that a difference in outcome was masked by high local mortality. In addition, the control used in the trial was not the standard of care in the US, where less toxic lipid-based amphotericin formulations are typically used,^2^ though to our knowledge there are no RCTs evaluating this regimen.^9^ In addition, to our knowledge there are no clinical data evaluating the AMBITION regimen in resource-rich settings.

Los Angeles General Medical Center is a large safety net hospital (80% Medicaid-covered, 5% uninsured) in downtown Los Angeles, providing care for a primarily underserved patient population. The burden of prolonged hospitalization is particularly challenging for this medical center’s patient population, many of whom cannot financially afford to forgo work or other responsibilities for 14 days. Thus, after the AMBITION trial was published in 2022, we purposefully migrated our care of CM in people with HIV at Los Angeles General Medical Center to the trial’s regimen. We conducted a pre-post cohort study to assess the clinical outcomes, adverse events, and health care utilization of the CM treatment regimen of the AMBITION trial compared with traditional induction among people with HIV. As the study took place in a safety net public hospital system, there was particular focus on the potential advantages of outpatient-based therapy for patients with financial and social barriers to care.

## Methods

### Study Design

Following publication of the AMBITION trial,^4^ our institution transitioned to primarily using the new protocol for CM in people with HIV: a single dose of liposomal amphotericin (10 mg/kg) plus 14 days of fluconazole (1200 mg/d) and flucytosine (100 mg/kg/d). This change was generally adopted, but there was no formalized training, and individual treatment plans were at the discretion of practitioners. In this cohort study, we retrospectively conducted electronic medical record (EMR) review to assess the outcomes of the change, comparing people with HIV treated with the standard regimen (14 days of lipid-based amphotericin at 3-5 mg/kg/d plus flucytosine [100 mg/kg/d]) from August 1, 2020, through July 31, 2022, with those treated with the AMBITION protocol from August 1, 2022, through July 31, 2024. The University of Southern California institutional review board approved this study with a waiver of informed consent because the data were deidentified. This study adhered to the Strengthening the Reporting of Observational Studies in Epidemiology (STROBE) reporting guideline.

### Patient Identification and Inclusion and Exclusion Criteria and Definitions

Potential patients were identified from the EMR database by positive cerebrospinal fluid (CSF) cryptococcal antigen (CRAG) test results during the study period, and their records were reviewed to determine eligibility. Patients were included if they had a confirmed HIV diagnosis and a positive CSF CRAG test result and were initiated on antifungal induction therapy.

Exclusion criteria included age less than 18 years, alternative diagnosis prompting discontinuation of induction therapy, transfer to an out-of-network hospital during induction, and death within 48 hours of admission. Patients during the intervention period that did not receive the AMBITION protocol were excluded from the primary analysis but examined separately to assess for selection bias. Additional HIV-associated complications or opportunistic infections were not excluded.

Per-protocol analysis required completion of the initial induction regimen. Patients that died during induction were excluded. Outpatient regimens were considered complete if documentation indicated the patient had taken medications as directed.

Race and ethnicity, ascertained from the EMR based on self-reported identification, were included in the analysis because racial disparities are an important social determinant of health. Categories were Asian, Black, Hispanic, and White.

### Outcome Measures

The primary outcome was a composite end point reflecting recurrence-free and severe adverse event–free 90-day survival. Prioritized secondary outcomes included completion of induction and consolidation therapy, 90-day and 1-year CM recurrence, hospital length of stay, 90-day readmission, rate of patient-directed discharge, and mortality during induction and at 30 days, 90 days, and 1 year. Recurrence was defined as antifungal induction by the treating practitioners due to clinical concern for active CM (regardless of culture results). Stable housing was defined as residing in a personal or family residence or medically adjacent support facility (eg, skilled nursing facility, recuperative housing). Patient-directed discharge was defined as occurring against medical advice in hospital discharge records. For all outcomes, only patients with follow-up data at the specified end point were included in the analysis.

Adverse events of interest included cytopenia, metabolic and electrolyte abnormalities, hepatotoxicity, infusion reaction, line complication, or drug allergy during antifungal therapy. Monitoring occurred at the discretion of the treating teams. A severe infusion reaction was defined as infusion-related hypotension requiring vasopressor support. Only grade 3 or 4 laboratory analyte abnormalities, as defined by Common Terminology Criteria for Adverse Events, version 5.0, were included.

### Statistical Analysis

We examined cohort differences of categorical variables using the χ^2^ test. Continuous variables were analyzed using the Mann-Whitney *U* test. Analysis was performed using KyPlot statistical software, version 6.0 (KyensLab Inc). For CSF CRAG levels, results exceeding the upper limit of quantification were right censored at the threshold of 1:2560. Statistical significance was defined as 2-sided *P* < .05.

## Results

### Study Population

The initial EMR query elicited 123 total positive CSF CRAG results during the study period. Fifty-nine (48.0%) occurred during the baseline control period (2020-2022), of which 7 (11.9%) were excluded as repeat samples during the same episode or hospitalization. Another 26 (44.1%) were excluded due to not having active CM (13 [50.0%]), absence of HIV (5 [19.2%]), transfer to an outside hospital during induction (4 [15.4%]), admission for an unrelated reason after recently successfully completing CM induction (3 [11.5%]), and patient-directed discharge prior to initiating antifungal therapy (1 [3.8%]). The remaining 26 patients (50.0%) constituted the control-period intention-to-treat population. An additional 9 patients in the baseline control period (15.3%) were excluded from the per-protocol analysis due to deviations from the initial regimen (7 patient-directed discharges [77.8%] and 2 deaths [22.2%] during induction) (Figure).

**Figure.**
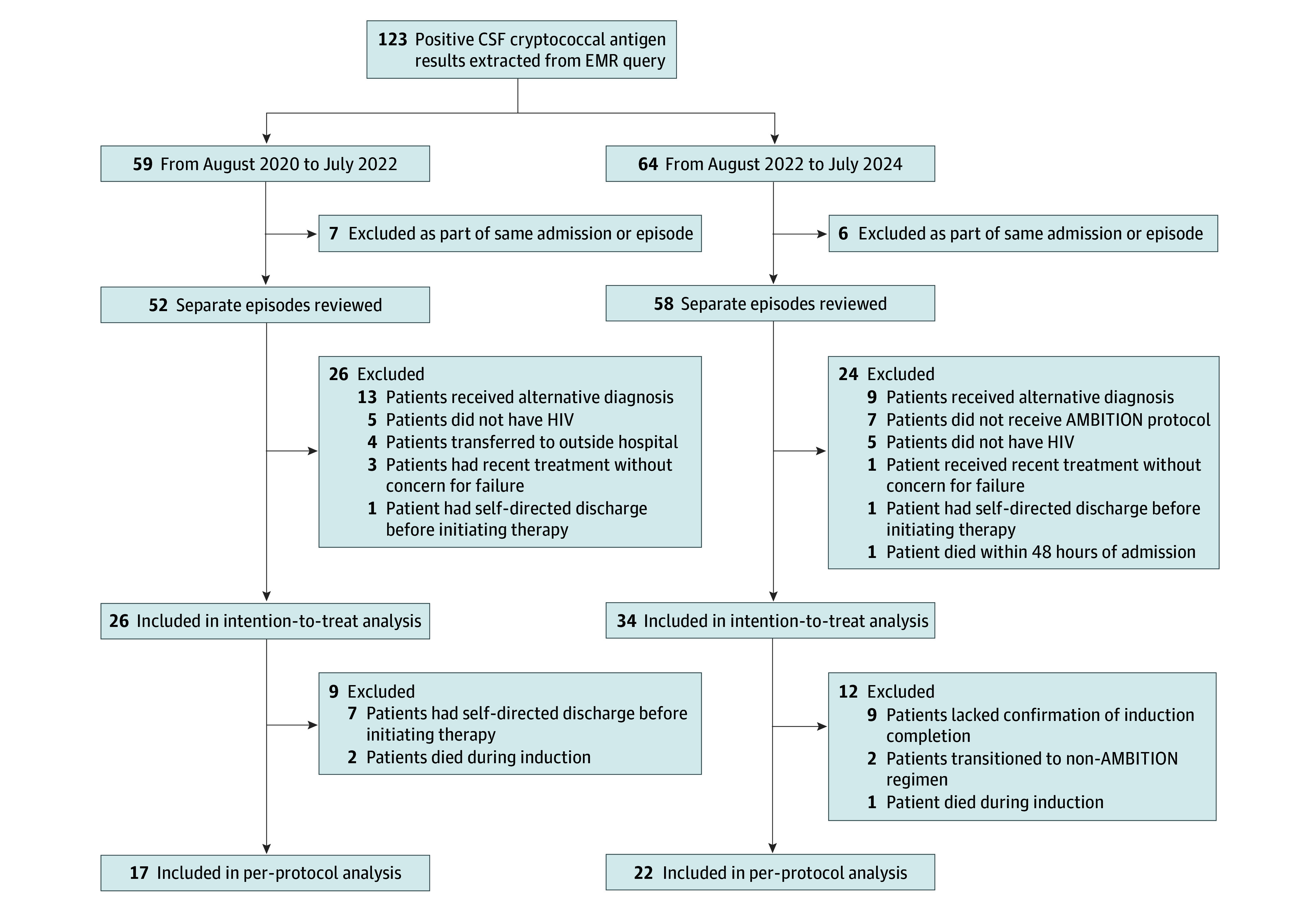
Flowchart of Screening, Exclusion, and Analysis CSF indicates cerebrospinal fluid; EMR, electronic medical record.

During the intervention period (2022-2024), 64 (52.0%) of the 123 positive CSF CRAG results were identified, of which 6 (9.4%) were excluded as repeat samples during same episode or hospitalization. Another 24 patients with positive CSF CRAG results (37.5%) were excluded due to not having active CM (9 [37.5%]), not receiving the AMBITION protocol (7 [29.2%]), not having HIV (5 [20.8%]), admission for an unrelated reason after recently successfully completing CM induction (1 [4.2%]), patient-directed discharge prior to initiating therapy (1 [4.2%]), and death within 48 hours of admission (1 [4.2%]). The remaining 34 patients (53.1%) constituted the intervention intention-to-treat population. An additional 12 patients in the intervention period (18.8%) were excluded from the per-protocol analysis due to deviations from the initial regimen (9 [75.0%] were without confirmed completion, 2 [16.7%] transitioned to alternative induction, and 1 [8.3%] died during induction) (Figure).

A total of 60 intention-to-treat patient episodes (26 [43.3%] in the control and 34 [56.7%] in the intervention period) were ultimately included in the study. Five patients (8.3%) were female, 54 (90.0%) were male, and 1 (1.7%) was transgender; median age was 40 years (IQR, 34-51 years). One patient (1.7%) was Asian, 7 (11.7%) were Black, 45 (75.0%) were Hispanic, 2 (3.3%) were White, and in 5 episodes (8.3%), patient race and ethnicity were not documented. Baseline characteristics of the intention-to-treat cohort were similar in both arms, including median CD4+ cell count, housing instability, presenting symptoms, and CSF findings (Table 1). In the per-protocol analysis (n = 39), there was a statistically significant increased rate of culture-confirmed CM in the control vs intervention period (17 of 17 patients [100%] vs 17 of 22 [77.3%]; *P* = .04), but this was not present in the intention-to-treat analysis.

**Table 1.** Baseline Characteristics

Baseline characteristic	Patient episodes^a^					
	Intention to treat (n = 60)			Per protocol (n = 39)		
	Control period (n = 26)	Intervention period (n = 34)	*P* value	Control period (n = 17)	Intervention period (n = 22)	*P* value
Age, median (IQR), y	40 (36-51)	41 (33-50)	.85	38 (33-52)	37 (31-47)	.46
Gender						
Female	2 (7.7)	3 (8.8)	.88	1 (5.9)	2 (9.1)	.71
Male	23 (88.5)	31 (91.2)	.73	15 (88.2)	20 (90.9)	.78
Transgender	1 (3.8)	0	.25	1 (5.9)	0	.25
Race and ethnicity						
Asian	0	1 (2.9)	.38	0	0	>.99
Black	2 (7.7)	5 (14.7)	.40	2 (11.8)	2 (9.1)	.78
Hispanic	20 (76.9)	25 (73.5)	.76	13 (76.5)	18 (81.8)	.68
White	1 (3.8)	1 (2.9)	.85	0	1 (4.5)	.37
Not documented	3 (11.5)	2 (5.9)	.43	2 (11.8)	1 (4.5)	.40
HIV data						
New diagnosis	6 (23.1)	9 (26.5)	.76	4 (23.5)	6 (27.3)	.79
Current ART	4 (15.4)	8 (23.5)	.43	3 (16.7)	3 (13.6)	.73
Active opportunistic infection or HIV-related malignant neoplasm	5 (19.2)	5 (14.7)	.64	2 (11.8)	4 (18.2)	.58
CD4+ cell count, median (IQR), cells/mm^3^	24.5 (12.7-54.7)	16.5 (8.0-42.5)	.24	30.0 (19.0-74.0)	16.5 (8.0-41.0)	.09
Comorbidities						
Chronic kidney disease	0	1 (2.9)	.38	0	0	>.99
Cirrhosis	0	1 (2.9)	.38	0	0	>.99
Active malignant neoplasm	2 (7.7)	1 (2.9)	.40	1 (5.9)	1 (4.5)	.85
Injection drug use	0	1 (2.9)	.38	0	1 (4.5)	.37
Housing status						
Housing instability	11 (42.3)	14 (41.2)	.93	5 (29.4)	5 (22.7)	.64
Incarcerated	2 (7.7)	1 (2.9)	.40	1 (5.9)	1 (4.5)	.85
Signs and symptoms						
Headache	22 (84.6)	33 (97.1)	.08	13 (76.5)	21 (95.5)	.08
Altered mental status	8 (30.8)	13 (38.2)	.55	5 (29.4)	8 (36.4)	.65
Intubation	1 (3.8)	5 (14.7)	.16	0	2 (9.1)	.20
Vasopressor requirement	1 (3.8)	2 (5.9)	.72	0	1 (4.5)	.37
Seizure	2 (7.7)	1 (2.9)	.40	1 (5.9)	1 (4.5)	.85
Fever	10 (38.5)	16 (47.1)	.51	8 (47.1)	10 (45.5)	.92
Hydrocephalus, No./total No. (%)	1/26 (3.8)	1/33 (3.0)	.86	0	0	>.99
Fungemia, No./total No. (%)	12/23 (52.2)	21/33 (63.6)	.39	6/15 (40.0)	12/22 (54.5)	.38
CSF findings						
Opening pressure, median (IQR), cm H_2_O	26 (21-33)	21 (15-31)	.42	26 (19-33)	19 (17-28)	.29
White blood cell count, median (IQR), /µL	7 (2-37)	12 (3-55)	.55	18 (2-109)	5 (3-55)	.58
Cryptococcal antigen, titer, median (IQR)	≥1:2560 (1:160 to ≥2560)	≥1:2560 (1:160 to ≥2560)	.75	≥1:2560 (1:160 to ≥2560)	1:1280 (1:80 to ≥2560)	.77
Positive stain result	23 (88.5)	26 (76.5)	.23	16 (94.1)	16 (72.7)	.08
Positive culture result	25 (96.2)	29 (85.3)	.16	17 (100)	17 (77.3)	.04
LPs, median (IQR), No.	3 (2-3)	2 (1-3)	.14	3 (2-4)	2 (1-2)	.06
Invasive CSF drain	1 (3.8)	4 (11.8)	.27	1 (5.9)	2 (9.1)	.71

Abbreviations: ART, antiretroviral therapy; CSF, cerebrospinal fluid; LP, lumbar puncture.

SI conversion factor: To convert white blood cell count to × 10^9^/L, multiply by 0.001.

^a^ Unless otherwise indicated, data are presented as number (percentage) of patient episodes (or as number out of total number [percentage] to reflect missing data).

Loss to follow-up occurred in 15 patients by the 90-day primary outcome, representing 25.0% of all intention-to-treat episodes. Data at each time point were missing for 4 control episodes (15.4%) and 5 intervention episodes (14.7%) at 30 days (*P* = .94), 6 control episodes (23.1%) and 9 intervention episodes (26.5%) at 90 days (*P* = .76), and 8 control episodes (30.8%) and 17 intervention episodes (50.0%) at 1 year (*P* = .13).

Of 58 episodes reviewed in the intervention period, 7 patients (12.1%) received the daily amphotericin-based regimen during the intervention period at practitioners’ discretion and were not included in the primary analysis. A sensitivity analysis was performed comparing those patients with the intervention arm to assess for selection bias (eTable 1 in Supplement 1). The baseline characteristics were comparable, and there was a statistically significant lower median CSF white blood cell count in the control than in the intervention arm (1/µL [IQR, 1-6/µL] vs 12/µL [IQR, 3-55/µL]; *P* = .050) (to convert to × 10^9^/L, multiply by 1.0). Otherwise, there were no significant differences between the groups.

### Intention-to-Treat Outcomes

In the intention-to-treat analysis, the composite outcome was achieved in 7 of 20 control patients (35.0%) and 19 of 25 intervention participants (76.0%) (*P* = .006). Ninety-day mortality occurred in 2 of 20 control patients (10.0%) and 1 of 25 intervention patients (4.0%) (*P* = .42). Ninety-day recurrence occurred in 1 of 18 control patients (5.6%) and 0 of 24 intervention patients with available data (*P* = .24).

Seventeen control patients (65.4%) and 24 intervention patients (70.6%) completed induction (*P* = .67). Four patients in the intervention cohort (11.8%) were transitioned to a daily amphotericin regimen (eTable 2 in Supplement 1). All 17 control patients who completed induction remained hospitalized for the entirety of induction; of the 24 who completed induction in the intervention cohort, 17 (70.8%) finished outpatient. Five patients in the intervention arm (14.7%) were lost to follow-up during induction, with unknown completion status.

Control patients were admitted for a median of 19 days (IQR, 11-26 days), compared with 7 days (IQR, 4-12 days) in the intervention arm (*P* < .001). Patient-directed discharges occurred in 9 control patients (34.6%), compared with 4 (11.8%) in the intervention arm (*P* = .03). Stable housing at discharge was arranged for 15 of 24 surviving, nontransferred control patients (62.5%), compared with 27 of 32 surviving, nontransferred patients in the intervention arm (84.4%) (*P* = .06). Consolidation was confirmed complete in 7 control patients (26.9%) and 18 intervention patients (52.9%) (*P* = .04). Additional outcome data are summarized in Table 2. Circumstances of recurrences and deaths are provided in eTables 3 and 4 in Supplement 1.

**Table 2.** Primary and Secondary Outcomes

Outcome	Patient episodes^a^					
	Intention to treat (n = 60)			Per protocol (n = 39)		
	Control (n = 26)	Intervention (n = 34)	*P* value	Control (n = 17)	Intervention (n = 22)	*P* value
Composite outcome^b^	7/20 (35.0)	19/25 (76.0)	.006	4/13 (30.8)	15/18 (83.3)	.003
All-cause mortality						
During induction	2/26 (7.7)	1/34 (2.9)	.40	NA	NA	NA
30 d	2/22 (9.1)	1/29 (3.4)	.40	0/14 (0)	0/21 (0)	NA
90 d	2/20 (10.0)	1/25 (4.0)	.42	0/13 (0)	0/18 (0)	NA
1 y	3/18 (16.7)	3/18 (16.7)	>.99	1/12 (8.3)	1/14 (7.1)	.91
Recurrence of cryptococcal meningitis						
90 d	1/18 (5.6)	0/24 (0)	.24	0/13 (0)	0/18 (0)	NA
1 y	6/17 (35.3)	4/17 (23.5)	.45	4/13 (30.8)	1/13 (7.7)	.14
Confirmed by culture	4/6 (66.7)	3/4 (75.0)	.78	3/4 (75.0)	1/1 (100)	.58
Antifungal status at recurrence						
Incomplete induction	2/6 (33.3)	3/4 (75.0)	.20	NA	NA	NA
Incomplete consolidation	2/6 (33.3)	0/4 (0)	.20	3/4 (75.0)	0/1 (0)	.17
Complete consolidation						
Not taking maintenance antifungal therapy	1/6 (16.7)	1/4 (25.0)	.75	0/4 (0)	1/1 (100)	.03
Taking maintenance antifungal therapy	1/6 (16.7)	0/4 (0)	.39	1/4 (25.0)	0/1 (0)	.58
Induction status						
Complete	17 (65.4)	24 (70.6)	.67	17 (100)	22 (100)	NA
Hospital	17/17 (100)	7/24 (29.2)	<.001	17 (100)	5 (22.7)	<.001
Outpatient	0	17/24 (70.8)	<.001	0	17 (77.3)	<.001
Incomplete	9 (34.6)	5 (14.7)	.07	NA	NA	NA
Unknown	0	5 (14.7)	.04	NA	NA	NA
Consolidation complete	7 (26.9)	18 (52.9)	.04	7 (41.2)	16 (72.7)	.047
Induction duration, median (IQR), d	14 (12-21)	14 (11-14)	.40	15 (14-21)	14 (14-14)	.21
Amphotericin duration, median (IQR), d	14 (12-21)	1 (1-1)	<.001	15 (14-21)	1 (1-1)	<.001
Hospitalization, median (IQR), d	19 (11-26)	7 (4-12)	<.001	22 (18-26)	7 (3-10 )	<.001
Patient-directed discharge	9 (34.6)	4 (11.8)	.03	3 (17.6)	0	.04
Stable housing at discharge	15/24 (62.5)	27/32 (84.4)	.06	14/17 (82.4)	19/21 (90.5)	.73
90-d Readmission	6/24 (25.0)	8/33 (24.2)	.95	2 (11.8)	5 (22.7)	.38

Abbreviation: NA, not applicable.

^a^ Unless otherwise indicated, data are presented as number (percentage) of patient episodes (or as number out of total number [percentage] to reflect missing data).

^b^ Recurrence-free and severe adverse event–free 90-day survival.

Adverse events were documented in 16 control patients (61.5%) and 7 intervention patients (20.6%) (*P* = .001) (Table 3). Cytopenia and metabolic abnormalities were more common in the control population. No grade 3 kidney injury occurred in either arm. A higher median peak creatinine level was seen among control patients (1.22 mg/dL [IQR, 1.01-1.56 mg/dL]) compared with intervention patients (1.00 mg/dL [IQR, 0.81-1.37]) (*P* = .03) (to convert to µmol/L, multiply by 88.4), along with a larger median percentage creatinine change from admission in the control arm (52% [IQR, 29%-116%]) compared with the intervention arm (28% [IQR, 5%-67%]) (*P* = .02). There was no significant difference in rates of hepatotoxicity.

**Table 3.** Adverse Events

Adverse event	Patient episodes^a^					
	Intention to treat (n = 60)			Per protocol (n = 39)		
	Control (n = 26)	Intervention (n = 34)	*P* value	Control (n = 17)	Intervention (n = 22)	*P* value
Any	16 (61.5)	7 (20.6)	.001	12 (70.6)	4 (18.2)	<.001
Cytopenia^b^	11 (42.3)	3 (8.8)	.002	7 (41.2)	1 (4.5)	.005
Metabolic abnormalities^c^	9 (34.6)	4 (11.8)	.03	7 (41.2)	3 (13.6)	.051
Creatinine level, median (IQR), mg/dL						
Admission	0.80 (0.65-0.88)	0.77 (0.63-0.97)	.94	0.78 (0.64-0.88)	0.72 (0.63-0.86)	.70
Peak	1.22 (1.01-1.56)	1.00 (0.81-1.37)	.03	1.21 (1.00-1.53)	0.89 (0.75-1.12)	.02
Change, %	52 (29-116)	28 (5-67)	.02	55 (42-116)	20 (5-56)	.01
Liver injury^d^	0	1 (2.9)	.38	0	1 (4.5)	.37
Severe infusion reaction^e^	1 (3.8)	0	.25	0	0	>.99
Line complication	0	0	>.99	0	0	>.99
Drug allergy	0	0	>.99	0	0	>.99

SI conversion factor: To convert creatinine to µmol/L, multiply by 88.4.

^a^ Data are presented as number (percentage) of patient episodes unless otherwise indicated.

^b^ Defined as hemoglobin level less than 8 g/dL (to convert to g/L, multiply by 10.0), platelet count less than 50 × 10^3^/µL (to convert to × 10^9^/L, multiply by 1.0), and/or white blood cell count less than 2 × 10^3^/µL (to convert to × 10^9^/L, multiply by 1.0).

^c^ Defined as creatinine level more than 3 times the upper limit of normal, potassium level less than 3 mEq/L (to convert to mmol/L, multiply by 1.0), sodium level less than 130 mEq/L (to convert to mmol/L, multiply by 1.0), calcium level less than 7 mg/dL (to convert to mmol/L, multiply by 0.25), magnesium level less than 0.9 mg/dL (to convert to mmol/L, multiply by 0.4114), and/or phosphorous level less than 2 mg/dL (to convert to mmol/L, multiply by 0.323).

^d^ Defined as aminotransferase level more than 5 times the upper limit of normal and/or total bilirubin level more than 3 times the upper limit of normal.

^e^ Defined as hypotension requiring vasopressor support.

### Per-Protocol Outcomes

In the per-protocol analysis, the composite outcome was met in 4 of 13 control episodes (30.8%) and 15 of 18 intervention episodes (83.3%) (*P* = .003). There were no 90-day deaths or recurrences in any patient who completed induction therapy.

All control patients remained hospitalized for the entirety of induction; in contrast, 17 of 22 intervention patients (77.3%) completed induction outpatient. Control patients were admitted for a median of 22 days (IQR, 18-26 days), compared with 7 days (IQR, 3-10 days) in the intervention cohort (*P* < .001). Patient-directed discharges occurred in 3 control patients (17.6%) compared with 0 intervention patients (*P* = .04). Stable housing at discharge was arranged for 14 of 17 surviving, nontransferred control patients (82.4%), compared with 19 of 21 surviving, nontransferred intervention patients (90.5%) (*P* = .73). Consolidation was confirmed complete in 7 control patients (41.2%) and 16 intervention patients (72.7%) (*P* = .047).

Adverse events were documented in 12 control patients (70.6%) and 4 intervention patients (18.2%) (*P* < .001) (Table 3). Cytopenia and metabolic abnormalities were more common in the control population. No grade 3 kidney injury occurred in either arm. A higher peak creatinine level was seen among the control population (1.21 mg/dL [IQR, 1.00-1.53 mg/dL]) compared with intervention patients (0.89 mg/dL [IQR, 0.75-1.12 mg/dL]) (*P* = .02), along with a larger percentage creatinine change from admission among the control group (55% [IQR, 42%-116%]) compared with intervention patients (20% [IQR, 5%-56%]) (*P* = .01). There was no significant difference in rates of hepatotoxicity.

## Discussion

In this pre-post study, we found that people with HIV with CM had better recurrence- and severe adverse event–free 90-day survival when receiving the AMBITION protocol compared with control patients who received a guideline-concordant, daily lipid-based amphotericin regimen. The improved outcome was primarily driven by decreased adverse effects, specifically metabolic abnormalities and cytopenia. There was no statistically significant difference in mortality or recurrence at any of the assessed time intervals.

These results are consistent with those seen in the AMBITION RCT.^4^ A similar rate and distribution of grade 3-4 adverse effects were seen in our study’s control cohort and the AMBITION control arm. However, in our study, fewer total adverse events were noted in the intervention cohort than in the AMBITION trial (50% vs 20.6%). The higher adverse event rate in the AMBITION trial appears to have been driven by the “other” category, with the present study showing similar rates of cytopenia, metabolic abnormalities, and hepatotoxicity.

Notably, a much higher intention-to-treat mortality rate was seen in the AMBITION trial: the all-cause death rate at 10 weeks was 24.8% to 28.7%, compared with 4.0% to 10.0% at 90 days in the present study. The AMBITION findings are consistent with a preceding RCT based in Africa, which showed an even worse 10-week mortality rate of 35.1% to 39.7%.^10^ However, our study’s results are similar to those previously reported in high-resource settings.^2,11,12,13^ The different outcomes likely reflect resource availability and access to care, with possible contribution of practice patterns.^12^ Additionally, higher rates of loss to follow-up in studies performed in resource-rich settings may have led to an underestimation of true mortality rate if a substantial proportion of the missing patients died. Nevertheless, our results suggest that the AMBITION protocol can be successfully applied to a resource-rich setting.

One of the most important findings of this study was that the AMBITION protocol appeared to be more patient-centered. Patient-directed discharges (against medical advice) were significantly less frequent in the intervention arm, and more patients were able to arrange stable housing and establish long-term care to complete consolidation therapy. This likely reflects better alignment between patient preferences and medical recommendations, allowing for improved therapeutic alliance and reduced care interruptions. Although few prior studies have discussed patient-directed discharges in CM, our study’s rates were higher than the previously reported 2% to 9%.^14,15^ As a safety net hospital, many of our institution’s patients have competing financial and social responsibilities, making prolonged hospitalizations particularly challenging. By aligning treatment recommendations with patient goals and preferences, this approach appears to enhance engagement into clinic and allow for more collaborative care. In addition, the shorter hospitalizations and less frequent monitoring allow for resource reallocation to other high-risk conditions and populations.

The primary issue that we have experienced introducing the AMBITION protocol is ensuring patient adherence outside the hospital. The regimen requires frequent dosing and many pills (6 fluconazole 200-mg tablets daily and 12 to 20 flucytosine 500-mg capsules divided into 4 times daily), which presents a significant challenge to patients. Social and financial barriers should be taken into consideration in determining which patients can be treated in the outpatient setting, and additional resources should be provided when able. Additionally, close clinical monitoring is more difficult (for example, assessing for increased intracranial pressure requiring therapeutic lumbar puncture); thus, outpatient management optimally occurs in patients with low-risk features. Our hospital has a protocolized out-of-hospital acute care program (“Safer at Home”) with remote monitoring and streamlined urgent care access,^16^ which is increasingly used for higher-risk patients with CM. However, even patients requiring admission for the duration of induction for social or medical reasons can be safely treated with the AMBITION protocol. In addition, loss to follow-up is a significant issue for our patient population, and we employ a robust case management and social work team to assist patients established in HIV primary care clinics.

Future research directions include prospective and randomized studies in resource-rich settings and assessment of interventions to improve patient monitoring and medication adherence in outpatient settings. Evaluation of patients’ subjective experience and quality-of-life measures would also be instructive to help guide patient-centered care.

### Limitations

The primary limitation of our study is its pre-post nature, making it prone to various forms of bias. As patients during the intervention period were not required to receive the AMBITION protocol, it is possible that sicker patients received the daily amphotericin-based regimen, excluding them from the primary analysis and predisposing the intervention arm toward better outcomes. However, additional analysis showed similar baseline characteristics (eTable 1 in Supplement 1). Additionally, due to more frequent outpatient treatment, the AMBITION arm received less monitoring, which may have underestimated adverse events. Our study was also limited by loss to follow-up, which occurred in 23.1% and 26.5% of the control and intervention arms at 90 days. To accommodate for this, outcomes were analyzed only for patients with data available at the specified end point. An additional limitation is the impact of changes to individual induction regimens, which occurred in 4 patients (11.8%) in the intervention arm, highlighting the importance of the per-protocol analysis. In addition, comparison with a historic instead of a concurrent control group may result in differences that reflect changes over time rather than impact of the AMBITION protocol; however, there were no other appreciated alterations in CM management over this period. Our findings were overall consistent with those seen in the AMBITION RCT.

## Conclusions

In this cohort study, the findings of the large AMBITION RCT, which was conducted in an LMIC setting, were recapitulated in a clinical experience at a large safety net hospital in the US. Use of the AMBITION protocol compared with guideline-compliant treatment was associated with a marked reduction in severe adverse events with no difference in mortality or CM recurrence. The ability to transition induction to the outpatient setting after initial inpatient management in carefully selected and closely monitored patients may allow for more patient-centered care, as reflected by decreased rates of patient-directed discharges and improved completion of consolidative therapy. These findings support broader adoption of the AMBITION protocol.

## Notes

Supplement 1. **eTable 1.** Comparison of Patients Receiving AMBITION vs Daily Amphotericin (August 2022 to July 2024) **eTable 2.** Reasons for Change in Regimen (August 2022 to July 2024) **eTable 3.** Cause of Death in Intention-to-Treat Arm **eTable 4.** Antifungal Status at Recurrence in Intention-to-Treat Arm

Supplement 2.
Data Sharing Statement
