# Supplementary material for: Single-Dose Liposomal Amphotericin Plus Fluconazole and Flucytosine for Cryptococcal Meningitis at a US Public Hospital
Source: JAMA Netw Open. 2026 Jan 21;9(1):e2553552. doi: 10.1001/jamanetworkopen.2025.53552 (PMC12824767; doi:10.1001/jamanetworkopen.2025.53552)
Supplement: Supplement 1. — eTable 1. Comparison of Patients Receiving AMBITION vs Daily Amphotericin (August 2022 to July 2024) eTable 2. Reasons for Change in Regimen (August 2022 to July 2024) eTable 3. Cause of Death in Intention-to-Treat Arm eTable 4. Antifungal Status at Recurrence in Intention-to-Treat Arm [file jamanetwopen-e2553552-s001.pdf]

Supplementary Online Content

Clark D, Barranco-Trabi J, Goo I, et al. Single-dose liposomal amphotericin plus fluconazole and flucytosine for cryptococcal meningitis at a US public hospital. *JAMA Netw Open*. 2026;9(1):e2553552. doi:10.1001/jamanetworkopen.2025.53552

eTable 1. Comparison of Patients Receiving AMBITION vs Daily Amphotericin (August 2022 to July 2024)

eTable 2. Reasons for Change in Regimen (August 2022 to July 2024)

eTable 3. Cause of Death in Intention-to-Treat Arm

eTable 4. Antifungal Status at Recurrence in Intention-to-Treat Arm

This supplementary material has been provided by the authors to give readers additional information about their work.

© 2026 Clark D et al. *JAMA Network Open*.

Comparison of Patients Receiving AMBITION vs Daily Amphotericin (August 2022 – July 2024)

During the later time period (August 2022 – July 2024), seven patients were initiated on a daily amphotericin-based regimen instead of the AMBITION protocol. The decision for induction regimen was at the treating providers' discretion. For three patients (43%), the patient was not felt to be a good candidate for an outpatient regimen from an adherence perspective (however, the AMBITION protocol can be completed as an inpatient). One patient (14%) was admitted with a relapse of CM after previously receiving AMBITION. No documentation regarding decision was provided in three cases (43%).

eTable 1. Comparison of Patients Receiving AMBITION vs Daily Amphotericin (August 2022 – July 2024)

Intention to Treat<br>Baseline Characteristics	Daily Amphotericin (n = 7)	AMBITION (n = 34)	*P* value
**Reason for Daily Amphotericin**			
**Poor candidate for outpatient regimen**	3 (43%	N/A	
**Recurrence after receiving AMBITION**	1 (14%)	N/A	
**Not documented**	3 (43%)	N/A	
**Age (IQR)**	44 (36-50.5)	40.5 (32.5-50)	0.77
**Gender**			
**Male (%)**	6 (86%)	31 (91%)	0.66
**Female (%)**	1 (14%)	3 (8.8%)	0.66
**Transgender (%)**	0%	0%	
**Race**			
**Hispanic (%)**	5 (71%)	25 (74%)	0.91
**Black (%)**	0 (0%)	5 (15%)	0.28
**Asian (%)**	0 (0%)	1 (2.9%)	0.65
**White (%)**	0 (0%)	2 (2.9%)	0.65
**Other (%)**	1 (14%)	0%	0.03
**Not documented (%)**	1 (14%)	2 (5.9%)	0.44
**HIV Status**			
**New Diagnosis (%)**	3 (43%)	9 (26%)	0.39
**Current ART (%)**	1 (14%)	8 (24%)	0.59
**Active OI or HIV-related malignancy (%)**	3 (43%)	5 (15%)	0.09
**Median blood CD4+ cell count (IQR)**	22 (13.5 - 36)	16.5 (8.0 - 42.5)	0.72
**Comorbidities**			
**Chronic Kidney Disease**	0 (0%)	1 (2.9%)	0.65
**Cirrhosis**	0 (0%)	1 (2.9%)	0.65
**Active Malignancy**	0 (0%)	1 (2.9%)	0.65
**Injection Drug Use**	0 (0%)	1 (2.9%)	0.65
**Housing**			
**Housing instability (%)**	3 (43%)	14 (41%)	0.93
**Incarcerated (%)**	0 (0%)	1 (2.9%)	0.65
**Signs/Symptoms**			
**Headache (%)**	6 (86%)	33 (97%)	0.20
**Altered Mental Status (%)**	1 (14%)	13 (38%)	0.22
**Intubation (%)**	0 (0%)	5 (15%)	0.28
**Pressor requirement (%)**	0 (0%)	2 (5.9%)	0.44
**Seizure (%)**	0 (0%)	1 (2.9%)	0.65

© 2026 Clark D et al. *JAMA Network Open*.

**Fever (%)**	1 (14%)	16 (47%)	0.11
**Hydrocephalus (%)[a]**	0/5 (0%)	1/33 (3.0%)	0.69
**Fungemia (%)[b]**	5/7 (71%)	21/33 (64%)	0.69
**CSF findings**			
**Opening Pressure, cm H2O (IQR)**	20 (14 – 30)	21 (15 -31)	0.49
**Median CSF WBC (IQR)**	1 (1 – 6)	12 (3 – 55)	0.05
**Median CSF Cryptococcal Antigen (IQR)**	≥1:2560 (1:640 - ≥2560)	≥1:2560 (1:160 - ≥2560)	0.56
**CSF Stain Positive (%)**	6 (86%)	26 (76%)	0.59
**CSF Culture Positive (%)**	6 (86%)	29 (85%)	0.98
**Number of LPs (IQR)**	2 (1-4.5)	2 (1-3)	0.93
**Invasive Drain (%)**	1 (14%)	4 (12%)	0.85

[a] Only patients with brain imaging included
[b] Only patients with blood culture data included

© 2026 Clark D et al. *JAMA Network Open*.

Reasons for Change in Regimen (August 2022 to July 2024)

Four patients initially receiving the AMBITION protocol were transitioned to alternative regimen. The reason for change of the four patients was documented as clinical worsening requiring external ventricular drain (EVD), lack of improvement in mental status despite placement of EVD, persistently positive CSF cultures on day two of treatment, and inability to tolerate oral medications due to nausea (placed on amphotericin monotherapy since unable to tolerate flucytosine – no IV fluconazole administered). One patient initially receiving daily amphotericin and flucytosine was transitioned to high-dose fluconazole and flucytosine regimen due to provider preference (not included in primary analysis).

eTable 2. Reasons for Change in Regimen (August 2022 to July 2024)

	Daily Amphotericin (n = 7)	AMBITION (n = 34)	*P* value
**Regimen Changed**	1 (14%)	4 (12%)	0.85
**Inadequate Response**	0	1 (2.9%)	0.65
**Clinical Worsening**	0	1 (2.9%)	0.65
**Persistent Positive Cultures**	0	1 (2.9%)	0.65
**Unable to Take Oral Medication**	0	1 (2.9%)	0.65
**Provider Preference**	1 (14%)	0	0.03

© 2026 Clark D et al. *JAMA Network Open*.

Cause of Death in Intention-to-Treat arm

In the intention-to-treat control, three patients died within one year. Two patients died during induction, on day 4 and day 10, with both deaths attributed to cryptococcal meningitis. One patient died >90 days due to disseminated *Mycobacterium avium* complex infection and unknown brain mass.

In the intention-to-treat intervention arm, three patients died within one year. One patient died on day 3, attributed to cryptococcal meningitis. The other patients died >90 days, one due to primary CNS lymphoma and the other due to advanced Kaposi sarcoma.

eTable 3. Cause of Death in Intention-to-Treat Arm

**Intention to Treat**	**Control (n = 26)**	**Intervention (n = 34)**	**P value**
**Cause of Death (n)**	3	3	
**Cryptococcus**	2 (67%)	1 (33%)	0.41
**Other Opportunistic Infection**	1 (33%)	0 (0%)	0.27
**Malignancy**	0 (0%)	2 (67%)	0.08

© 2026 Clark D et al. *JAMA Network Open*.

Antifungal Status at Recurrence in Intention-to-Treat arm

In the intention-to-treat control, six patients were diagnosed with recurrent cryptococcal meningitis within one year. Two (33%) had not completed induction; two (33%) had completed induction, but not consolidation and one (17%) had completed induction and consolidation but stopped maintenance antifungal therapy. One patient (17%) was treated for recurrence despite being actively on fluconazole maintenance therapy. We defined recurrence as prompting re-induction by treating providers, so this was included despite higher suspicion for immune reconstitution inflammatory syndrome).

In the intention-to-treat intervention arm, four patients were diagnosed with recurrent cryptococcal meningitis within one year. Three (75%) had not completed induction. Of these, one had left as patient-directed discharge and did pick up antifungal or follow-up in clinic. The other two were intentional discharges, but were unable to adhere to induction regimen. One patient (25%) completed induction and consolidation, but subsequently lost to follow-up and off antifungal therapy.

eTable 4. Antifungal Status at Recurrence in Intention-to-Treat arm

**Intention to Treat**	**Control (n = 26)**	**Intervention (n = 34)**	***P* value**
**Antifungal Status at Recurrence (n)**	6	4	
**Incomplete induction**	2 (33%)	3 (75%)	0.20
**Completed induction, but not consolidation**	2 (33%)	0 (0%)	0.20
**Complete induction/consolidation, but off antifungal**	1 (17%)	1 (25%)	0.75
**Completed induction/consolidation and on maintenance**	1 (17%)	0 (0%)	0.39

© 2026 Clark D et al. *JAMA Network Open*.
