# Supplementary material for: Single-Dose Liposomal Amphotericin Plus Fluconazole and Flucytosine for Cryptococcal Meningitis at a US Public Hospital
Source: JAMA Netw Open. 2026 Jan 21;9(1):e2553552. doi: 10.1001/jamanetworkopen.2025.53552 (PMC12824767; doi:10.1001/jamanetworkopen.2025.53552)
Supplement: Supplement 2. — Data Sharing Statement [file jamanetwopen-e2553552-s002.pdf]

Data Sharing Statement

Clark. Single-Dose Liposomal Amphotericin Plus Fluconazole and Flucytosine for Cryptococcal Meningitis at a US Public Hospital. *JAMA Netw Open*. Published January 21, 2026. doi:10.1001/jamanetworkopen.2025.53552

Data
Data available: Yes
Data types: Deidentified participant data
How to access data: dclark2@dhs.lacounty.gov
When available: With publication

Supporting Documents
Document types: None

Additional Information
Who can access the data: Researchers whose proposed use of the data has been approved
Types of analyses: For research purposes
Mechanisms of data availability: With investigator support
